# Type 2 Diabetes and Chronic Kidney Disease: An Opportunity for Pharmacists to Improve Outcomes

**DOI:** 10.3390/jcm13051367

**Published:** 2024-02-28

**Authors:** Joshua J. Neumiller, Wendy L. St. Peter, Jay H. Shubrook

**Affiliations:** 1 Department of Pharmacotherapy, College of Pharmacy and Pharmaceutical Sciences, Washington State University, Spokane, WA 99210, USA; 2 Department of Pharmaceutical Care & Health Systems, University of Minnesota, Minneapolis, MN 55455, USA; stpet002@umn.edu; 3 Department of Clinical Sciences and Community Health, Touro University California, Vallejo, CA 94592, USA; jshubroo@touro.edu

**Keywords:** renal insufficiency, chronic, diabetes mellitus, type 2, pharmacist, heart and kidney protection, multidisciplinary care

## Abstract

Chronic kidney disease (CKD) is an important contributor to end-stage kidney disease, cardiovascular disease, and death in people with type 2 diabetes (T2D), but current evidence suggests that diagnosis and treatment are often not optimized. This review examines gaps in care for patients with CKD and how pharmacist interventions can mitigate these gaps. We conducted a PubMed search for published articles reporting on real-world CKD management practice and compared the findings with current recommendations. We find that adherence to guidelines on screening for CKD in patients with T2D is poor with particularly low rates of testing for albuminuria. When CKD is diagnosed, the prescription of recommended heart–kidney protective therapies is underutilized, possibly due to issues around treatment complexity and safety concerns. Cost and access are barriers to the prescription of newer therapies and treatment is dependent on racial, ethnic, and socioeconomic factors. Rates of nephrologist referrals for difficult cases are low in part due to limitations of information and communication between specialties. We believe that pharmacists can play a vital role in improving outcomes for patients with CKD and T2D and support the cost-effective use of healthcare resources through the provision of comprehensive medication management as part of a multidisciplinary team. The Advancing Kidney Health through Optimal Medication Management initiative supports the involvement of pharmacists across healthcare systems to ensure that comprehensive medication management can be optimally implemented.

## 1. Introduction

Chronic kidney disease (CKD) associated with type 2 diabetes (T2D) is a growing health problem, with a prevalence of approximately 40% in adults with T2D in the USA [1,2]. The treatment landscape for CKD associated with T2D (referred to here as T2D and CKD) is continually evolving, as demonstrated by US Food and Drug Administration (FDA) approvals of kidney protective agents in this field since 2020 [3,4,5,6]. Treatment guidelines have been updated accordingly, although evidence suggests that recommendations are not translating to clinical practice, resulting in suboptimal diagnosis and treatment of affected patients [7,8].

T2D and CKD is correlated with high morbidity, mortality, and poor quality of life [9]. It is the largest contributor to end-stage kidney disease (ESKD) worldwide and is associated with an increased risk of cardiovascular disease (CVD) and other microvascular complications in patients with T2D [9,10,11]. Despite this, recognition of T2D and CKD by healthcare professionals (HCPs) and patient awareness of this condition remain unacceptably low [12].

Multiple mechanisms contribute to the kidney pathophysiology of T2D and CKD [13]. Stimulation of the renin–angiotensin–aldosterone system (RAAS) increases the production of angiotensin II and vasoconstriction of the efferent arteriole, leading to increased glomerular pressure and filtration, albuminuria, and nephropathy [13]. Additionally, increased glucose reabsorption by the sodium–glucose cotransporter-2 (SGLT-2) in the proximal tubule decreases solute delivery to the macula densa and results in vasodilation of the afferent arteriole, also leading to increased glomerular pressure and hyperfiltration [14]. Another mechanism that contributes to CKD is mineralocorticoid receptor overactivation by aldosterone, resulting in inflammation and fibrosis [15,16]. Finally, hyperglycemia increases oxidative stress, inflammation, and fibrosis via various metabolic pathways contributing to kidney disease pathology [13]. Kidney damage associated with these mechanisms is evident through albuminuria and a reduced glomerular filtration rate (GFR), which can be estimated from serum creatinine levels (eGFR). Albuminuria and eGFR are both independent predictors of the risk of CVD and CKD-related events and death in patients with T2D [17], and both are used to stage CKD and to inform treatment decisions related to heart–kidney protection [8,18].

**Figure 1:**
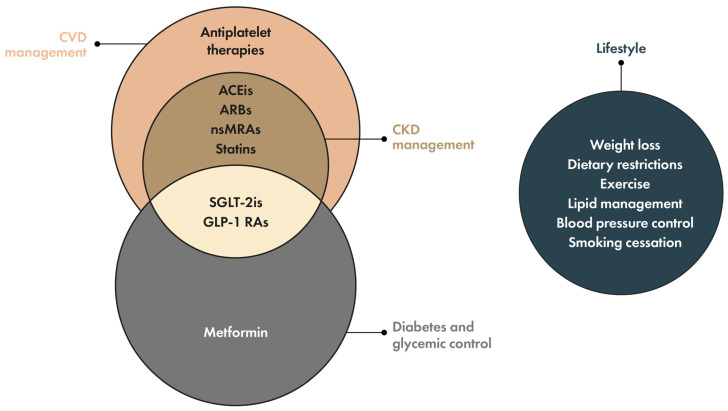
Treatment classes for diabetes, cardiovascular risk, and kidney protection, and lifestyle interventions for the multifactorial management of patients with T2D and CKD [8,18,20,21,22]. Abbreviations used: ACEis, angiotensin-converting enzyme inhibitors; ARBs, angiotensin II receptor blockers; CKD, chronic kidney disease; CVD, cardiovascular disease; GLP-1 RAs, glucagon-like peptide-1 receptor agonists; nsMRAs, nonsteroidal mineralocorticoid receptor antagonists; SGLT-2is, sodium–glucose cotransporter-2 inhibitors; T2D, type 2 diabetes.

A systematic approach to screening for CKD in patients with T2D is needed to ensure timely diagnosis and initiation of treatment, which are essential to preserve kidney function and reduce progression to ESKD, development of CVD, and death [5,19]. The 2023 American Diabetes Association (ADA) Standards of Care and the 2022 Kidney Disease: Improving Global Outcomes (KDIGO) guidelines for diabetes management in CKD recommend early diagnosis and multifactorial management of patients with T2D and CKD (Figure 1), including lifestyle interventions, such as dietary restrictions and exercise, glycemic control, blood pressure management, and lipid-lowering measures, as well as specific interventions for kidney protection, which will form the focus of this review [8,18,20,21,22]. Dietary measures no longer need to restrict proteins and recommend a standard protein intake of 0.8 g/kg bodyweight for people with T2D and CKD not receiving dialysis. Plant-based proteins along with a focus on vegetables, fruits, whole grains, fiber, unsaturated fats, and nuts are recommended, accompanied by limitations on processed meats and refined carbohydrates [18]. Lifestyle modifications, such as these dietary recommendations, together with exercise, smoking cessation, and weight management, form the foundation for the management of kidney–heart risk factors in all patients with T2D and CKD. Some of the recommended agents exhibit kidney protective effects independent of glycemic control and should be initiated regardless of glucose levels in patients with T2D and CKD [8].

Despite these treatment guidelines, many patients with T2D and CKD receive suboptimal care, demonstrated not only by the underutilization of recommended medications in clinical practice but also by racial and ethnic disparities in the extent to which treatment guidelines are followed [7]. Studies have shown that Black patients are less likely to receive newer diabetes medications, including SGLT-2 inhibitors (SGLT-2is) and glucagon-like peptide-1 receptor agonists (GLP-1 RAs), than White patients [23,24]. This is particularly pertinent given the increased prevalence of diabetes and hypertension, and younger average age at diabetes diagnosis, in non-Hispanic Black adults compared with non-Hispanic White adults, and the faster decline in eGFR in young Black versus White adults [2,25,26,27].

All members of the multidisciplinary team (MDT) managing patients with T2D and CKD are responsible for bridging these gaps in care. In the USA, primary care physicians (PCPs) and endocrinologists generally oversee the management of patients with T2D and CKD, with potential nephrologist intervention as kidney disease progresses [28]. Pharmacists, nurses, and dietitians also play an integral role in MDTs for patients with these multisystem diseases [18], to support multifactorial management, through lifestyle interventions, such as dietary restrictions and exercise, glycemic control, and kidney protection. Late referrals to nephrologists may mean that the progression of CKD cannot be slowed, and therefore early involvement of members across the team is key to ensuring that patients can access appropriate care in a timely manner.

Pharmacists are particularly well placed to address many of the barriers to optimal care provision for patients with T2D and CKD, such as low awareness and implementation of treatment guidelines by HCPs and the challenges of polypharmacy for both HCPs and patients [7]. Pharmacists could offer point-of-care testing for serum creatinine and urine albumin-to-creatinine ratio (UACR) to aid the identification of CKD in patients with T2D, improve care coordination across the MDT, and provide comprehensive medication management (CMM). In various studies, this has demonstrated many benefits for patients with chronic diseases, including improved clinical outcomes, patient safety, patient and provider satisfaction, and cost-effectiveness of care [29,30,31,32,33,34,35,36]. 

The objectives of this review article are to explore the gaps in current care for T2D and CKD, with a focus on kidney protection, and to discuss best practices, as recommended by treatment guidelines, highlighting the role of pharmacists in bridging identified gaps in care.

## 2. Comparison of Current Clinical Management for T2D and CKD Versus Best Practice

### 2.1. CKD Screening and Diagnosis

CKD is usually asymptomatic in the early stages, so regular screening is recommended in patients with T2D to ensure timely diagnosis and treatment initiation. The ADA and KDIGO recommend screening at least annually for CKD by assessing both urinary albumin and eGFR in all patients with T2D, regardless of treatment [8,37]. 

Disparities have been demonstrated between screening recommendations and clinical practice. A US study of >1.5 million patients with T2D found that less than half of patients had both serum creatinine and urine albumin measurements during a one-year follow-up period [38]. Even fewer patients had a UACR test compared with a serum creatinine test (43.3% vs. 84.8%), indicating selective adherence to screening guidelines by HCPs [38]. Early changes in kidney function can typically be detected by increases in albuminuria before changes in eGFR [8]. Therefore, a lack of albuminuria testing is likely to delay CKD diagnosis and the initiation of effective treatment. A pilot study showed that albuminuria testing rates can be significantly increased by implementing systematic testing with relatively simple changes to workflow [39]. 

The ADA recommends a clinical CKD diagnosis based on persistent albuminuria (UACR ≥ 30 mg/g) and/or sustained reduction in eGFR (<60 mL/min/1.73 m^2^), in the absence of other primary causes of kidney damage [8]. Similarly, KDIGO defines a CKD diagnosis as the presence of any of the following signs for >3 months: eGFR < 60 mL/min/1.73 m^2^, UACR ≥ 30 mg/g, or other markers of kidney disease [5,18]. CKD progression is defined as a decline in the eGFR category (G1–G5), and a certain drop is a decline in the eGFR category accompanied by a ≥25% drop in eGFR from baseline [40].

**Figure 2:**
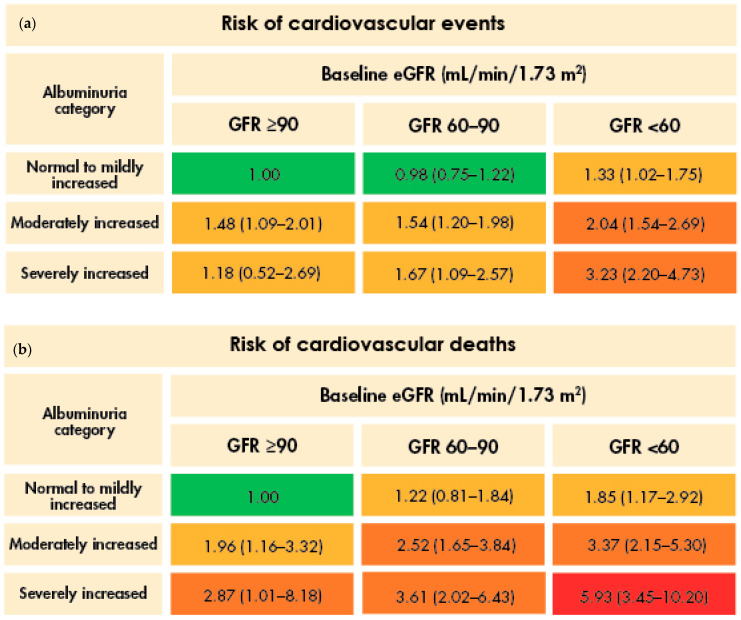

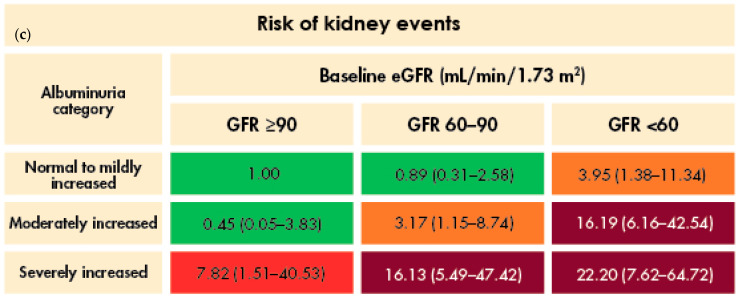
The combined effect of albuminuria and eGFR on the risk of cardiovascular events (**a**) and cardiovascular deaths (**b**), and kidney events (**c**), in patients with T2D [17]. The risk is color-coded, with green for values ≤ 1, yellow for values > 1 and ≤2, orange for values > 2 and ≤5, red for values > 5 and ≤10, and burgundy for values > 10. Higher values indicate an increased risk of events. Abbreviations used: eGFR, estimated glomerular filtration rate; GFR, glomerular filtration rate; T2D, type 2 diabetes.

The ADA-recommended albuminuria threshold for initiating treatment following a diagnosis of T2D and CKD is a UACR of ≥30 mg/g, even in patients with normal-to-high eGFR (≥90 mL/min/1.73 m^2^) [8]. This is because albuminuria is associated with an increased risk of CKD progression, kidney failure, CVD, and death in patients with T2D (Figure 2) [17,18]. Patients who reach the treatment threshold require multifactorial management, including specific measures for kidney protection [8].

### 2.2. Angiotensin-Converting Enzyme Inhibitors (ACEis)/Angiotensin II Receptor Blockers (ARBs)

ACEis and ARBs are an established standard of care for patients with T2D and CKD [5,18]. These agents inhibit the production and downstream signaling of angiotensin II, blocking the RAAS [18,21,41]. ACEis and ARBs work independently of reductions in blood pressure, reducing glomerular pressure; therefore, an associated increase in serum creatinine and decrease in eGFR can be expected with treatment [41]. Studies have shown that RAAS inhibition reduces albuminuria and the risk of CKD progression in patients with T2D [41,42,43]. However, there are potential adverse events (AEs) associated with RAAS inhibition. ACEis and ARBs may cause hypotension and hyperkalemia and lower eGFR to the extent that a reduced-perfusion acute kidney injury (AKI) may result. Other potential AEs include angioedema and dry cough, the risks of which are significantly higher with ACEis than ARBs [44,45]. Black patients have been reported to have a 4.5-fold increased risk of angioedema versus White patients when treated with ACEis, and the risk of dry cough is 2.5-fold higher for Asian patients than for White patients. Therefore, ARBs should be considered prior to ACEis in Black and Asian populations [46,47].

**Figure 3:**
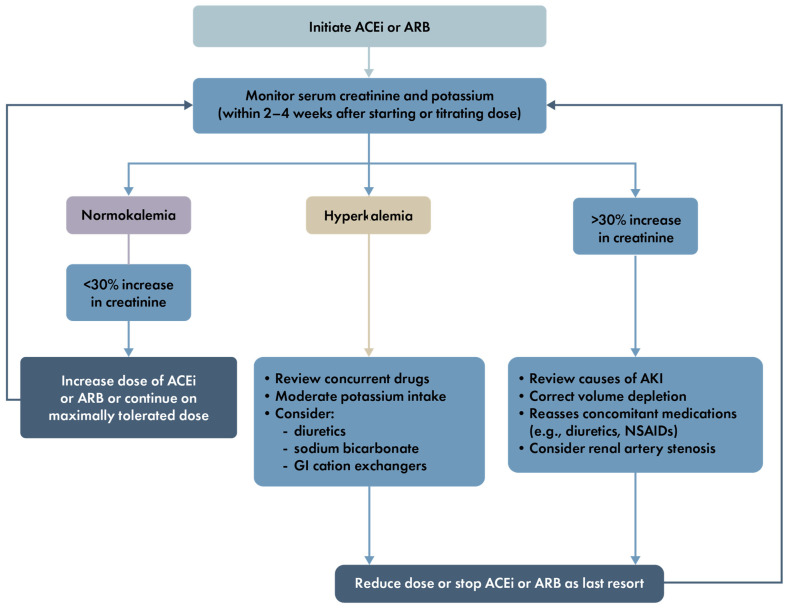
KDIGO recommendations for serum creatinine and potassium monitoring during ACEi/ARB treatment [18]. Abbreviations used: ACEi, angiotensin-converting enzyme inhibitor; AKI, acute kidney injury; ARB, angiotensin II receptor blocker; GI, gastrointestinal; KDIGO, Kidney Disease: Improving Global Outcomes; NSAID, non-steroidal anti-inflammatory drug. Reproduced from the KDIGO 2020 Clinical Practice Guideline for Diabetes Management in Chronic Kidney Disease, ref. [18] with permission from KDIGO.

The ADA and KDIGO guidelines recommend first-line management with either an ACEi or an ARB to reduce the risk or slow the progression of CKD in non-pregnant patients with diabetes, hypertension, UACR ≥ 30 mg/g, and/or eGFR < 60 mL/min/1.73 m^2^ [8,18,21,37]. The KDIGO guidelines recommend ACEis or ARBs for patients with diabetes, hypertension, and albuminuria, at the highest approved dose that is tolerated. ACEi or ARB treatment for the primary prevention of CKD in patients with T2D who have normal blood pressure, normal UACR, and normal eGFR is not recommended [8]. However, KDIGO recommends that ACEis or ARBs may be considered for normotensive patients with diabetes and albuminuria because a RAAS blockade reduces the severity of albuminuria, which is strongly correlated with the risk of kidney failure [18]. When ACEis, ARBs, or diuretics are used, both the ADA and KDIGO recommend regular monitoring of serum creatinine and potassium levels and that a RAAS blockade should not be discontinued for minor increases (≤30%) in serum creatinine in the absence of volume depletion [8]. KDIGO guidelines specifically suggest that serum creatinine and potassium should be monitored within 2–4 weeks of starting or titrating an ACEi or ARB dose (Figure 3) [18]. A recent update to the ADA guidelines recommends monitoring within 1–2 weeks of starting an ACEi or ARB [48], which is likely more prudent given how quickly these agents can impact on kidney function. Regardless, monitoring of blood pressure, serum potassium, and serum creatinine should be standard practice after initiating treatment or titrating the dose of these agents.

Multiple studies have assessed ACEi and ARB use in clinical practice. The Center for Kidney Disease Research, Education and Hope (CURE-CKD) registry includes over 2.6 million adults and children in the USA who have CKD or are at risk of developing CKD [49]. Data from this registry show that only 25% of adult patients (75,504/300,157) with CKD, diabetes/pre-diabetes, and hypertension were receiving a RAAS inhibitor [50]. This was supported by a cross-sectional US study that reported ACEi/ARB use in only 36% of patients with CKD from 2012 to 2014 [51]. Premature discontinuation of ACEi and ARB treatment has also been demonstrated. A retrospective study of 53,912 patients with CKD found that over 50% of patients discontinued ACEi or ARB therapy within 5 years of initiation, with more advanced CKD stage at initiation significantly correlated with increased discontinuation rates [52]. Interestingly, a UK study found that patients with kidney disease had a greater risk of treatment discontinuation than patients prescribed ACEis for other indications [53]. Hyperkalemia is a common reason for ACEi/ARB discontinuation and patients with CKD may be at particular risk if urinary potassium excretion is impaired. A real-world study of patients with heart failure and/or CKD showed that the risk of a serious heart and/or kidney event was significantly higher at 6 months in patients who discontinued or down-titrated ACEis/ARBs following a hyperkalemia event versus those who maintained or up-titrated their dose [54]. These findings emphasize the importance of managing hyperkalemia effectively and the need to optimize ACEi/ARB dosing following an episode of hyperkalemia as recommended by treatment guidelines [18].

### 2.3. SGLT-2is

SGLT-2is are an established treatment for glycemic management in patients with T2D, lowering glucose by reducing proximal tubule glucose reabsorption and increasing urinary glucose excretion [5,20,55]. Studies have shown that SGLT-2is reduce the risk of CKD progression and CVD in T2D compared with placebo when added to an optimized RAAS blockade [56,57,58,59,60]. The kidney protection associated with SGLT-2i treatment is largely independent of glycemic control and may be attributed to reduced RAAS activation, reduced glomerular pressure and hyperfiltration, inhibition of inflammation and fibrosis, and weight loss [5,61,62]. The clinical implications of these treatment effects include weight loss, improved blood pressure control, reduced albuminuria, and eGFR preservation [61]. The kidney protection conferred by weight loss and improved blood pressure is greater in mild to moderate CKD than in severe CKD because these effects lessen as eGFR declines [18].

The non-glycemic kidney-protective effects of SGLT-2is were supported by the DAPA-CKD trial, which found that the risk of a sustained ≥ 50% eGFR decline, ESKD, or death from kidney or cardiovascular causes was significantly lower with dapagliflozin treatment than with placebo, regardless of the presence or absence of diabetes [63]. In the CREDENCE trial, canagliflozin treatment reduced kidney outcomes (relative risk of ESKD, doubling of serum creatinine concentration, or death from kidney or cardiovascular events) by 30%, further highlighting the kidney protective effects of SGLT-2is [58]. Further supporting the kidney benefits of SGLT-2is, results from the EMPA-KIDNEY trial were recently reported demonstrating the benefit of empagliflozin treatment on a primary composite outcome of kidney disease progression (inclusive of progression to ESKD, a sustained decrease in eGFR to <10 mL/min/1.73 m^2^, a sustained decline in eGFR of ≥40% from baseline, or death from kidney causes) or cardiovascular death [64]. It should be noted that across these studies, most patients were also treated with an ACEi or ARB.

AEs associated with SGLT-2is may include polyuria, genital infections, and euglycemic diabetic ketoacidosis [5]. Other limitations of treatment may include the requirement for regular monitoring of blood pressure and volume status [5]. SGLT-2is may also be associated with muscle atrophy, and as T2D is a further risk factor for sarcopenia, patients should be assessed for other relevant risk factors, such as age, weight, and muscle mass when considering this type of treatment [65].

The ADA and KDIGO consensus report on diabetes management in CKD recommends prioritizing SGLT-2i agents with documented kidney or cardiovascular protective effects as first-line glycemic management, in addition to metformin, for patients with T2D and CKD with an eGFR ≥ 20 mL/min/1.73 m^2^ [37]. Moreover, SGLT-2is should be considered for heart–kidney protection regardless of patients’ glycemic control or the need for additional glucose lowering [37]. Once initiated, SGLT-2i treatment can be continued in the absence of intolerance (even if eGFR decreases to <20 mL/min/1.73 m^2^), until kidney replacement therapy commences [37].

Although SGLT-2i prescriptions for patients with CKD associated with diabetes have increased steadily over the past decade [66,67], overall SGLT-2i uptake in clinical practice remains low. A global study published in April 2022 of patients with T2D (DISCOVER) reported that 10.8% of patients were receiving an SGLT-2i, indicating that SGLT-2i use has not yet been optimized [68]. Reasons posited for this low uptake include the complexity of diabetes treatment, concerns around safety, clinical inertia, and cost [69]. Affordability and access to SGLT-2is are undoubtedly significant barriers, particularly in the US, where insurance coverage is a key factor. A recent NIH study of >80,000 US adults with diabetes showed that patients with health insurance were significantly more likely to receive an SGLT-2i and/or a GLP-1 RA than those without health insurance [70]. Even when insurance coverage exists, there may be significant out-of-pocket costs and access issues. An analysis of US Medicare beneficiaries showed that patients with T2D were significantly more likely to receive an SGLT-2i or a GLP-1 RA if these agents were included on less expensive formulary tiers (tiers 1–3) [71]. Guideline advocacy for first-line SGLT-2i use is likely to increase the duration of treatment and exacerbate cost issues [72].

Various studies have investigated racial and ethnic disparities in the prescription of diabetes medications. A US study focusing on SGLT-2i use in patients with diabetes found that SGLT-2is were less likely to be initiated in Black patients than in White patients (odds ratio: 0.93; 95% confidence interval [CI]: 0.91–0.95) [24]. Similarly, a secondary analysis of the US Look AHEAD trial identified racial and ethnic disparities in the initiation of newer diabetes medications, including SGLT-2is and GLP-1 RAs. Initiation of newer diabetes medications was lower in African American participants (hazard ratio [HR]: 0.81; 95% CI: 0.70−0.94) and American Indian/Alaskan Native participants (HR: 0.51; 95% CI: 0.26−0.99) versus White participants (*p* = 0.019). This correlation remained significant after adjusting for socioeconomic factors and was driven mainly by GLP-1 RA use [23]. Similar trends have been shown in real-word studies of patients with diabetes and CKD, where African American race was associated with lower use of SGLT-2is and GLP-1 RAs, higher use of sulfonylureas, and higher rates of hypoglycemia [73,74]. Racial and ethnic disparities in the prescription of diabetes medications are particularly relevant given the increased risk of T2D in Black African, Black Caribbean, and South Asian populations compared with White populations, and the increased risk of CKD progression to ESKD in African American patients, Hispanic patients, and Asian patients compared with White patients [75,76,77].

### 2.4. Mineralocorticoid Receptor Antagonists (MRAs)

MRAs inhibit the mineralocorticoid receptor, the overactivation of which results in inflammation and fibrosis, contributing to CKD progression in patients with T2D [15,16]. There are two classes of MRAs: steroidal MRAs (e.g., spironolactone and eplerenone) and nonsteroidal, selective MRAs (e.g., finerenone) [16]. Nonsteroidal MRAs are more selective for the mineralocorticoid receptor than steroidal MRAs and demonstrate balanced tissue distribution between the kidney and heart compared with steroidal MRAs, which accumulate in kidney tissue versus heart tissue [16].

Steroidal MRAs have demonstrated kidney and cardiovascular protective effects in clinical trials but are not recommended specifically for reducing CKD progression in patients with T2D, and their use is limited by the associated risk of hyperkalemia [8,16,78,79]. The KDIGO guidelines suggest that steroidal MRAs may be useful in patients with diabetes and resistant hypertension who have an eGFR > 45 mL/min/1.73 m^2^ and no history of hyperkalemia [3,18].

The lack of clear recommendations on steroidal MRA use in patients with T2D and CKD is reflected in clinical practice [8,50]. In a study published in 2019, 0.35% of adult patients (287/81,266) with CKD and diabetes/pre-diabetes in the CURE-CKD registry were receiving an MRA [50]. Furthermore, only 0.57% of adult patients (1702/300,157) with CKD, diabetes/pre-diabetes, and hypertension were receiving an MRA [50]. To date, no phase III clinical trial of a steroidal MRA has been conducted in patients with diabetes and CKD.

Currently, the only nonsteroidal MRA approved by the FDA and European Medicines Agency (EMA) for patients with T2D and CKD is finerenone [3]. Phase III clinical trial data from the FIDELIO-DKD study showed that, when added to an optimized RAAS blockade, finerenone significantly reduced the incidence of kidney failure, kidney death, or sustained ≥ 40% eGFR reduction from baseline versus placebo, demonstrating kidney-protective effects in patients with T2D and CKD [80]. The FIGARO-DKD phase III trial showed that finerenone also significantly reduced the incidence of a composite of cardiovascular death, myocardial infarction, stroke, and hospitalization for heart failure versus placebo in this cohort [81]. There are important safety considerations with finerenone treatment, including a risk of hyperkalemia; however, trial data indicated low hyperkalemia-related treatment discontinuation rates [80,81].

The pooled FIDELITY analysis comprising both FIDELIO-DKD and FIGARO-DKD phase III trial data has since been released, confirming that finerenone reduced the risk of clinically important kidney and cardiovascular outcomes versus placebo across a spectrum of CKD severity in patients with T2D [82]. The subsequent ADA and KDIGO 2022 consensus report recommends a nonsteroidal MRA with documented kidney or cardiovascular benefit (currently finerenone) for patients with T2D and CKD with an eGFR ≥ 25 mL/min/1.73 m^2^ and a UACR ≥ 30 mg/g despite a maximum tolerated dose of a RAAS inhibitor [37]. Candidates for finerenone therapy should have a normal serum potassium concentration (≤4.8 mmol/L) and undergo regular monitoring of serum potassium to mitigate the risk of hyperkalemia [18].

As finerenone was only granted FDA approval in July 2021, data are yet to be published on its use in clinical practice, but a prospective observational study is ongoing (FINE-REAL) [83]. A randomized study comparing finerenone in combination with the SGLT-2i empagliflozin versus either agent alone is also in progress (CONFIDENCE) [84]. Various other nonsteroidal MRAs are in clinical development, including apararenone, esaxerenone, KBP-5074, and AZD9977 [16].

### 2.5. GLP-1 RAs

GLP-1 RAs are an established treatment for glycemic management in patients with T2D and function partly by increasing glucose-dependent insulin secretion and inhibiting glucagon secretion [5,20,85]. Some studies show evidence of kidney protection with GLP-1 RA treatment in patients with T2D and CKD; however, the underlying mechanisms and their independence from glycemia remain under investigation [5,86,87,88,89,90]. GLP-1 RA treatment also reduces CVD risk compared with placebo in patients with T2D; however, associated AEs may include nausea, vomiting, and diarrhea [5,90,91,92]. There may also be a risk of hypoglycemia if a GLP-1 RA is used with insulin or insulin secretagogue medications [5].

The ADA guidelines recommend GLP-1 RA treatment to reduce cardiovascular risk in patients with T2D who have established CKD or CVD or multiple CVD risk factors [21]. Ongoing studies comparing GLP-1 RA treatment with placebo will provide further evidence on the effect of GLP-1 RAs on kidney function in patients with T2D and CKD [8,93]. KDIGO preferentially recommends the addition of a long-acting GLP-1 RA for glycemic control in patients with T2D and CKD who have not achieved individualized targets despite treatment with metformin and an SGLT-2i or those who are unable to tolerate these medications [18]. Improvements in glycemic control and reductions in eGFR decline with some GLP-1 RAs have also been reported in patients with moderate to severe CKD (eGFR of >15 mL/min/1.73 m^2^) [18,89]. Although guidelines suggest prioritizing injectable GLP-1 RAs with documented cardiovascular benefits (liraglutide, injectable semaglutide, and dulaglutide), both the ADA and KDIGO do not currently recommend GLP-1 RAs specifically for kidney protection in patients with T2D and CKD [8,18].

Despite no specific recommendations for GLP-1 RAs for kidney protection, secondary outcome data suggest potential kidney benefits [94]. Liraglutide resulted in reductions in persistent albuminuria, persistent doubling of serum creatinine, ESKD, and death due to kidney disease in the LEADER trial [88]. Injectable semaglutide reduced the rates of new or worsening CKD (SUSTAIN-6), and dulaglutide reduced the rates of severe albuminuria and eGFR decline (REWIND) [86,91]. Dulaglutide also slowed eGFR decline compared with insulin glargine (AWARD-7) [89]. Although the risk of hypoglycemia is generally low for GLP-1 RAs, dose modifications of concomitant insulin and/or sulfonylureas may be required [18]. Beyond glycemic control in T2D and CKD, the management of kidney function and CVD risk is complex and based on multiple patient factors.

Evidence suggests that although GLP-1 RA use has increased over time, these medications remain underutilized in clinical practice [68]. In a study published in 2019, only 0.23% of adult patients (183/81,266) with CKD and diabetes/pre-diabetes in the CURE-CKD registry were receiving a GLP-1 RA [50]. Of the adult patients with CKD, diabetes/pre-diabetes, and hypertension, 0.42% (323/300,157) were receiving a GLP-1 RA. This modest increase compared with patients without hypertension may be explained partly by the proven cardioprotective effects of GLP-1 RAs [90,91,92]. Greater uptake of GLP-1 RAs than SGLT-2is (0.23% vs. 0.10%, respectively) was also noted in patients with CKD and diabetes/pre-diabetes, which can likely be attributed to the more recent FDA approvals of SGLT-2is compared with GLP-1 RAs and the resultant hesitancy of physicians to prescribe medications with a smaller real-world evidence base [8,90].

**Figure 4:**
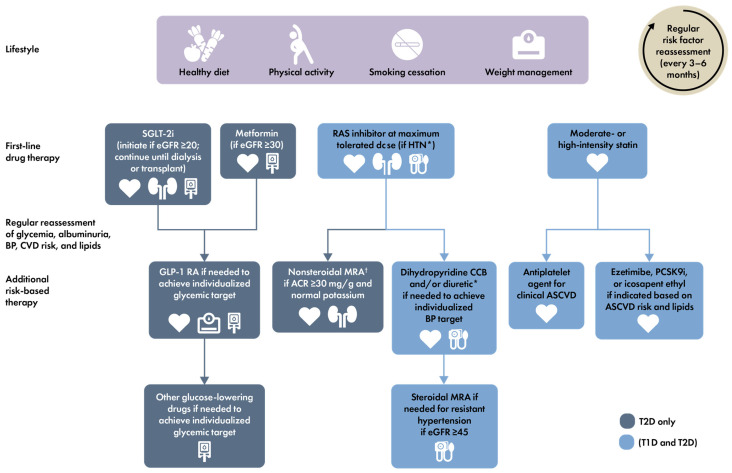
Holistic approach for improving outcomes in patients with diabetes and CKD [18]. Icons indicate the following benefits: BP cuff, BP-lowering; glucometer, glucose-lowering; heart, cardioprotection; kidney, kidney protection; scale, weight management. Estimated glomerular filtration rate (eGFR) is presented in units of mL/min/1.73 m^2^. * ACEi or ARB (at maximal tolerated doses) should be first-line therapy for hypertension when albuminuria is present. Otherwise, dihydropyridine calcium channel blocker or diuretic can also be considered; all three classes are often needed to attain BP targets. † Finerenone is currently the only ns-MRA with proven clinical kidney and cardiovascular benefits. Abbreviations used: ACEi, angiotensin-converting enzyme inhibitor; ACR, albumin-to-creatinine ratio; ARB, angiotensin II receptor blocker; ASCVD, atherosclerotic cardiovascular disease; BP, blood pressure; CCB, calcium channel blocker; CKD, chronic kidney disease; CVD, cardiovascular disease; eGFR, estimated glomerular filtration rate; GLP-1 RA, glucagon-like peptide-1 receptor agonist; HTN, hypertension; MRA, mineralocorticoid receptor antagonist; ns-MRA, nonsteroidal MRA; PCSK9i, proprotein convertase subtilisin/kexin type 9 inhibitor; RAS, renin–angiotensin system; SGLT-2i, sodium–glucose cotransporter-2 inhibitor; T1D, type 1 diabetes; T2D, type 2 diabetes. Reproduced from the KDIGO 2022 Clinical Practice Guideline for Diabetes Management in Chronic Kidney Disease, ref. [18] with permission from KDIGO.

A summary of treatment strategies for CKD in patients with T2D and CKD, including cardio- and kidney-protective therapies covered by this article, is shown in Figure 4.

### 2.6. Referral to a Nephrologist

As kidney disease progresses in patients with T2D, referral to a nephrologist may be required. The specific ADA-recommended referral criteria include an eGFR < 30 mL/min/1.73 m^2^, kidney disease of uncertain etiology, difficult management issues (including significant albuminuria despite blood pressure control), and/or rapidly progressing kidney disease [8]. The KDIGO 2012 guidelines recommend referral to a nephrologist when patients experience AKI or an abrupt sustained fall in GFR, GFR < 30 mL/min/1.73 m^2^, consistent albuminuria (UACR ≥ 300 mg/g), or CKD progression [40].

Interestingly, real-world evidence suggests that nephrologist referral guidelines are not implemented consistently in patients with CKD [95]. This may be explained by the findings of a qualitative study of 32 PCPs, which identified five key barriers to timely nephrologist referral and effective co-management of patients with CKD: a lack of timely adequate information exchange, limited access to nephrologists, poor working relationships with nephrologists, unclear delineation of roles and responsibilities between PCPs and nephrologists, and a lack of trust between patients and nephrologists [96].

## 3. Addressing Gaps in CKD Management

### 3.1. The MDT Approach to CKD Management

T2D and CKD is a multisystem disease associated with many potential complications and comorbidities [5]. The resultant need for an MDT covering both primary and specialist healthcare settings is well recognized and highlighted in the KDIGO guidelines [18]. KDIGO recommends that policymakers and institutional decision-makers implement team-based, integrated care for patients with T2D and CKD [18]. This is supported by a recommendation for comprehensive care delivered by physicians (PCPs, nephrologists, and cardiologists) and other members of the MDT (including pharmacists, nurses, and dietitians), to reduce the risks of CKD progression and CVD [18].

### 3.2. Opportunities for Pharmacists to Address the Gaps in Care for Patients with T2D and CKD

Pharmacists can optimize medication use, close critical gaps in CKD care, and improve health outcomes for patients with T2D and CKD by providing various services [5,34]. CMM is a consistent care process that can be delivered by pharmacists and members of the MDT to help patients achieve their treatment goals. CMM requires pharmacists to assess the indication (to determine its appropriateness), effectiveness, and safety of the drug, and the ability of the patient to medicate as intended (supporting increased adherence) for each medication and develop an individualized, evidence-based care plan for each patient. Further, as continuous glucose monitoring (CGM) devices develop, some linked to wearable insulin pumps, there is a growing need to support patients in selecting and understanding these devices to optimize and individualize disease control. The aims of the CMM approach are to improve population health, increase patient and provider satisfaction, and reduce per-capita healthcare costs; however, there are challenges to implementation in practice, including the consistent delivery of patient care and service replication by different pharmacists [97]. Although CGM can improve health outcomes, such as glycemic control, and quality of life, there is limited information on pharmacist-driven CGM interventions in community pharmacy settings, and educational, logistical, workflow, and financial barriers have been identified in the implementation this intervention [98].

**Figure 5:**
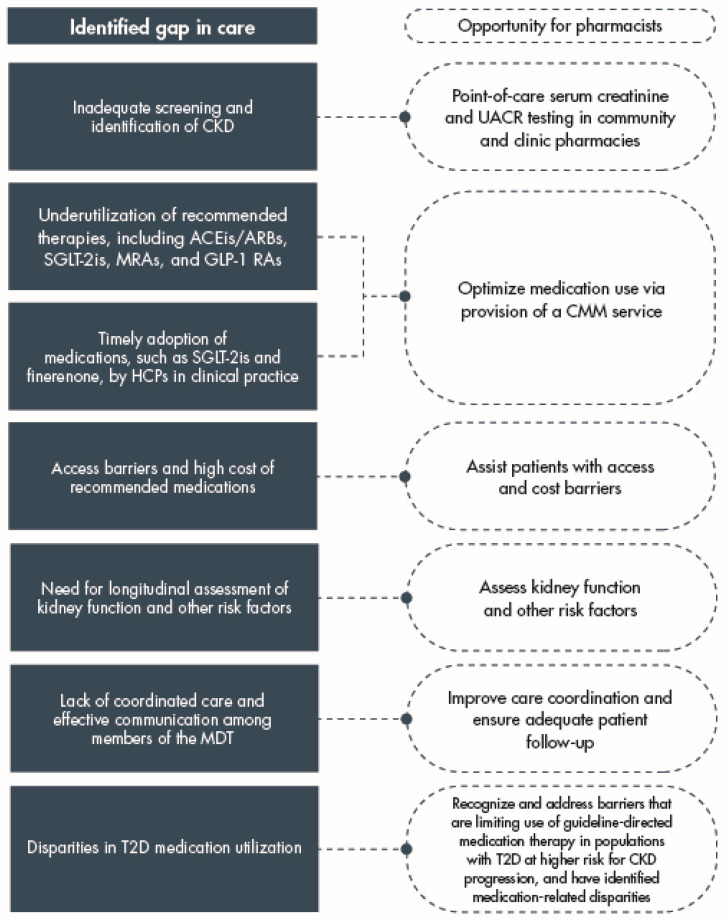
Key gaps in the management of patients with T2D and CKD and opportunities for pharmacists to address these barriers to optimal care provision [7,23]. Abbreviations used: ACEis, angiotensin-converting enzyme inhibitors; ARBs, angiotensin II receptor blockers; CKD, chronic kidney disease; CMM, comprehensive medication management; GLP-1 RAs, glucagon-like peptide-1 receptor agonists; HCP, healthcare professional; MDT, multidisciplinary team; MRAs, mineralocorticoid receptor antagonists; SGLT-2is, sodium–glucose cotransporter-2 inhibitors; T2D, type 2 diabetes; UACR, urine albumin-to-creatinine ratio.

Additional opportunities exist for pharmacists in the management of patients with T2D and CKD (Figure 5). For patients with T2D and CKD, there is a requirement to coordinate treatment of both diabetes and kidney dysfunction, as well as potential CVD and other comorbidities [8,18]. Pharmacists can play a vital role in resolving medication therapy problems, supporting patient health literacy, and ensuring that patients understand their potentially complex treatment strategies. Pharmacists also have the knowledge and skills to assist patients with cost barriers and access to medications, assess kidney function and risk factors for disease progression (such as glycemia, blood pressure, and lipid levels), and provide adequate follow-up that may be too time-consuming for a nephrologist to deliver [34]. Additionally, pharmacists have the potential to improve continuity of care and care coordination between different members of the MDT, which has been shown to reduce the risk of progression to ESKD in patients with T2D and CKD [34,97].

In recent years, there has been a shift toward value-based healthcare models, which are challenging healthcare providers to meet both efficiency and quality standards [99]. As part of the Advancing American Kidney Health initiative, the Centers for Medicare & Medicaid Services have developed value-based kidney care payment models that provide incentives for nephrology practices to improve outcomes in patients with CKD [100,101]. These new value-based care models have created opportunities for pharmacists to be integrated into nephrology and other practices to provide CMM for patients with T2D and CKD. The Advancing Kidney Health through Optimal Medication Management (AKHOMM; www.kidneymedicationmanagement.org (accessed on 19 February 2024)) initiative aims to ensure all people with CKD receive optimal medication management, by helping nephrology practices, value-based kidney care companies, and health systems implement CMM within their practices by incorporating pharmacists into their care teams to improve outcomes for patients with CKD [102]. Nephrology pharmacy practice and education standards have been developed and are being used to create a curriculum and training program for practicing pharmacists on providing CMM in patients with kidney diseases [103,104]. Furthermore, a learning and action collaborative is being established to help pharmacists and MDTs implement CMM in their practices.

As the most accessible members of the MDT, pharmacists are also in a position to address racial and ethnic disparities in the use of treatment guidelines, supporting health literacy and access to optimal care, and AKHOMM has initiatives to work with focus groups to better understand the needs of communities including African American patients with CKD [105].

### 3.3. The Value of Clinical Pharmacist Intervention in CKD

Through its different forms, clinical pharmacist intervention in the management of CKD has demonstrated added value for patients, healthcare teams, and healthcare systems [29]. Various studies have linked pharmacist intervention to improved clinical outcomes for patients with CKD, including improved hemoglobin control in patients with anemia and improved blood pressure control [30,106,107,108]. One study of 335 hypertensive patients with diabetes and/or CKD in the USA assessed the impact of pharmacist intervention on blood pressure control. Patients who received treatment recommendations from clinical pharmacists had a greater reduction in mean systolic blood pressure at 9 months compared with those who received the usual care (difference of 8.64 mm Hg; 95% CI: 4.49–12.8; *p* < 0.001) [30].

In addition to improved clinical outcomes, pharmacist intervention is associated with reduced medication errors and enhanced patient safety [31]. In a study of 136 kidney transplant recipients, participants who received pharmacist-led medication therapy management via a mobile health-based application experienced reductions in medication errors (incident risk ratio [IRR]: 0.39; 95% CI: 0.28–0.55; *p* < 0.001) and risk of Grade ≥ 3 AEs (IRR: 0.55; 95% CI: 0.30–0.99; *p* = 0.05) compared with the usual care and a return on investment of USD 4.30 for every USD 1 spent on the intervention [31,109].

Another potential benefit of pharmacist intervention is reduced healthcare utilization by patients. A 2-year randomized controlled trial assessing the impact of pharmacist intervention on hospitalization rates in 104 patients receiving hemodialysis found that patients in the intervention arm had fewer mean all-cause hospitalizations compared with patients receiving the standard of care (1.8 vs. 3.1; *p* = 0.02) [110]. Similarly, a study of 324 patients receiving hemodialysis reported reductions in the risk of unplanned hospital admissions (IRR: 0.73; 95% CI: 0.54–0.99; *p* = 0.047) and mean length of hospital stay (6.7 vs. 8.0 days; *p* < 0.001) for patients in the pharmacist intervention arm compared with the usual care [111]. Pharmacist intervention in the form of a CMM visit upon hospital discharge also reduced patient readmission rates within 30 days of discharge compared with no CMM visit (8.6% vs. 12.8%; *p* < 0.001) [112].

This reduction in healthcare utilization following pharmacist intervention has resulted in more cost-effective care for patients with CKD. A budget impact analysis of a high-risk Medicaid population after hospitalization found that those receiving pharmacist-led CMM services saved USD 2139 each, driven by a 32% reduction in hospital readmissions [35].

Other potential benefits of pharmacist intervention include improved patient and provider satisfaction rates. A survey of 119 primary care providers found that at least 87% of respondents strongly agreed or somewhat agreed that pharmacist integration into primary care resulted in reduced provider workload and emotional exhaustion, effective patient management, and improved achievement of patient treatment goals and quality measures [32].

Multiple studies have reported improvements in patient adherence to treatment following pharmacist interventions, such as CMM, patient education, and counseling; however, these studies vary in quality, and further evidence is required [29,113,114,115,116]. There is also a need for additional high-quality research on the impact of pharmacist intervention and team-based care specifically in patients with T2D and CKD [18,29]. Finally, there is an opportunity for pharmacists to improve CKD screening and diagnosis by offering point-of-care testing in community and clinic pharmacies.

## 4. Summary and Conclusions

T2D and CKD is a growing public health problem globally [1,2]. Although the treatment landscape is continually evolving, resulting in updates to treatment guidelines, adherence to recommendations remains low in clinical practice [8,18,37], demonstrated by suboptimal CKD screening in patients with T2D and consistent underutilization of ACEis/ARBs, SGLT-2is, MRAs, and GLP-1 RAs in eligible patients [7,8,49]. There are also significant racial and ethnic disparities in the application of treatment guidelines for diabetes and CKD, often with the highest-risk patients receiving a disproportionately low quality of care [22,24,117].

There is a need to adopt team-based diabetes healthcare models that integrate all members of the MDT, including pharmacists, nurses, and dietitians, to bridge the identified gaps in care [7,18]. Although all MDT members are responsible for improving the quality of care delivered to patients with T2D and CKD, pharmacists are particularly well suited to overcome some of the barriers to optimal medication management [34].

Interventions by clinical pharmacists, such as CMM and patient education, have the potential to optimize medication use and improve clinical outcomes, patient safety, patient and provider satisfaction, and the cost-effectiveness of care for patients with T2D and CKD [29,30,97]. Pharmacists may also be able to improve care coordination across the MDT, assist patients with cost barriers and access to medications, assess kidney function and risk factors for disease progression, and provide follow-up that may be too time-consuming for a nephrologist to deliver [34].

In conclusion, team-based care can meet the challenge of T2D and CKD. Clinical pharmacists should be fully integrated into the MDT to facilitate increased implementation of treatment guidelines, optimization of medication management, and improved patient outcomes. The AKHOMM initiative has been created to train practicing pharmacists on medication management in patients with CKD and to provide a learning and action collaborative to help implement pharmacist-provided CMM into practice.

## Data Availability

No new data were created or analyzed in this study. Data sharing is not applicable to this article.
